# Responses to the Hydrostatic Pressure of Surface and Subsurface Strains of *Pseudothermotoga elfii* Revealing the Piezophilic Nature of the Strain Originating From an Oil-Producing Well

**DOI:** 10.3389/fmicb.2020.588771

**Published:** 2020-12-04

**Authors:** Marie Roumagnac, Nathalie Pradel, Manon Bartoli, Marc Garel, Aaron A. Jones, Fabrice Armougom, Romain Fenouil, Christian Tamburini, Bernard Ollivier, Zarath M. Summers, Alain Dolla

**Affiliations:** ^1^Aix Marseille Univ., Université de Toulon, CNRS, IRD, MIO, Marseille, France; ^2^ExxonMobil Research and Engineering Company, Annandale, NJ, United States

**Keywords:** oil-reservoir, hydrostatic pressure, piezophile, chained cells, Pseudothermotoga, thermophilic anaerobes

## Abstract

Microorganisms living in deep-oil reservoirs face extreme conditions of elevated temperature and hydrostatic pressure. Within these microbial communities, members of the order *Thermotogales* are predominant. Among them, the genus *Pseudothermotoga* is widespread in oilfield-produced waters. The growth and cell phenotypes under hydrostatic pressures ranging from 0.1 to 50 MPa of two strains from the same species originating from subsurface, *Pseudothermotoga elfii* DSM9442 isolated from a deep African oil-producing well, and surface, *P. elfii* subsp. *lettingae* isolated from a thermophilic sulfate-reducing bioreactor, environments are reported for the first time. The data support evidence for the piezophilic nature of *P. elfii* DSM9442, with an optimal hydrostatic pressure for growth of 20 MPa and an upper limit of 40 MPa, and the piezotolerance of *P. elfii* subsp. *lettingae* with growth occurring up to 20 MPa only. Under the experimental conditions, both strains produce mostly acetate and propionate as volatile fatty acids with slight variations with respect to the hydrostatic pressure for *P. elfii* DSM9442. The data show that the metabolism of *P. elfii* DSM9442 is optimized when grown at 20 MPa, in agreement with its piezophilic nature. Both *Pseudothermotoga* strains form chained cells when the hydrostatic pressure increases, especially *P. elfii* DSM9442 for which 44% of cells is chained when grown at 40 MPa. The viability of the chained cells increases with the increase in the hydrostatic pressure, indicating that chain formation is a protective mechanism for *P. elfii* DSM9442.

## Introduction

The organisms growing preferentially under a hydrostatic pressure higher than the atmospheric pressure are called “piezophiles” (Zobell and Johnson, 1949; Yayanos, 1995). Most microorganisms characterized as psychrophilic to mesophilic piezophiles belong to the domain of *Bacteria* and have been isolated from deep marine environments. Except for a few bacterial isolates, thermophilic and hyperthermophilic piezophiles are found in the domain of *Archaea* and have been isolated mainly from deep-sea hydrothermal vents (Picard and Daniel, 2013; Jebbar et al., 2015). However, the rather limited number of piezophilic isolates [56 piezophiles isolated to date according to Cario et al. (2019)] fails to reflect the high diversity of microorganisms inhabiting subsea and subsurface ecosystems observed by cultivation-independent techniques. Improvement in cultivation at elevated pressures should provide access to species adapted to *in situ* pressure conditions and thus should result in the accumulation of more in-depth information on species inhabiting the deep biosphere (Tamburini et al., 2013; Cario et al., 2019; Garel et al., 2019).

It is known that high hydrostatic pressure impacts the activity of numerous key processes, leading to a drastic decrease in cell activity and eventually to the cell death of piezosensitive organisms (Bartlett, 2002; Simonato et al., 2006; Lauro and Bartlett, 2008; Tamburini et al., 2013). Piezophiles have developed various strategies to cope with elevated pressure to maintain cell integrity and function over a wide hydrostatic pressure range. According to the genera previously investigated, piezophilic adaptation requires either the modification of a few genes, a more profound reorganization of the genome, the fine-tuning of gene expression, or a stress-like physiological cell response. The most significant finding in studying the biochemistry of piezophiles is the discovery of *de novo* synthesis of polyunsaturated fatty acids (PUFAs), with their proportions reaching as much as 70% of the total fatty acids (FAs) in the membranes of these bacteria. The high proportion of PUFAs may increase the fluidity of the membrane at high hydrostatic pressure (Allen and Bartlett, 2002; Abe, 2013). The increase in the unsaturated FAs present in phospholipids has been also found in intracellular wax esters of a piezotolerant hydrocarbonoclastic bacterium (Grossi et al., 2010). A second major finding is the accumulation of solutes in the bacteria, which may play the expected role of a “piezolyte.” Like the mechanisms reported for microorganisms in response to osmotic or heat stresses, piezolytes may play the role of protein-stabilizing solutes. Accumulations of β-hydroxybutyrate and mannosyl-glycerate have been reported in *Photobacterium profundum* and the hyperthermophilic piezophilic archaeon *Thermococcus barophilus*, respectively (Martin et al., 2002; Cario et al., 2016). Similarly, glutamate has been proposed as a piezolyte in the piezophiles *Desulfovibrio hydrothermalis* and *Desulfovibrio piezophilus* (Amrani et al., 2014, 2016). Moreover, energy metabolism has been observed as one of the most important cellular process impacted in high-pressure adaptation (Le Bihan et al., 2013; Pradel et al., 2013). Enzymes produced by high pressure-adapted bacteria have been shown to exhibit higher activity under high-pressure conditions than at atmospheric pressure. In this context, the expression or the structure (for example, due to amino acid replacements) of enzymes may be modified in piezophilic microbes, favoring activity and stability in high-hydrostatic pressure conditions. For examples, a pressure-resistant terminal oxidase was discovered in *Shewanella violacea* (Tamegai et al., 2011); a piezophilic bioluminescent bacterium isolated at a 2,200 m water depth, *Photobacterium phosphoreum* ANT-2200, exhibits higher bioluminescence under high hydrostatic pressure than at atmospheric pressure (Martini et al., 2013) while no change at the transcriptional level of the light-emission involved genes can be detected (Tanet et al., 2019); and in the anaerobic sulfate-reducing bacterium *D. piezophilus*, transcriptomic and biochemical analyses have shown that metabolite cycling (H_2_/Formate) is an important mechanism required for energy conservation at high hydrostatic pressure (26 MPa; Pradel et al., 2013; Amrani et al., 2016). However, because these strategies have been identified by biochemical and genomic studies performed on cultured piezophilic strains, they yield a fragmented view on the adaptive mechanisms in piezophiles. One might expect that among the diverse and uncultured species of the deep biosphere, other unique and so-far unknown metabolic and physiological strategies to cope with elevated hydrostatic pressure are yet to be discovered (Cario et al., 2019).

Deep-oil reservoirs are inhabited by a wide range of thermophilic anaerobic microorganisms that have to face *in situ* high pressure (Cai et al., 2015). Studies of such microbes should lead to the discovery of unique adaptation strategies to these extreme conditions. While to date, the pressure within oil reservoirs (up to 50 MPa) has not been considered to preclude the development of microbes *in situ*, it is noted that it influences their physiological or metabolic properties (Magot et al., 2000). While some studies describing the oil microbial community have been carried out while respecting the *in situ* hydrostatic pressure of sampling (Kotlar et al., 2011; Lewin et al., 2014), the influence of high pressure has not been studied for any microorganisms inhabiting deep-oil reservoirs, while it should be considered as one of the critical environmental factors that shape oil reservoir biogeochemistry, and consequently, the inhabiting microbial community. Studying the effects of hydrostatic pressure on the development and physiology of these microorganisms is therefore of great interest for understanding the dynamics of the oil reservoir microbiome.

Both cultivation- and molecular-based investigations have demonstrated that members of the order *Thermotogales* are ubiquitous and predominant in global oil reservoirs (Magot et al., 2000; Magot, 2005; Ollivier and Cayol, 2005). Among them, representatives of the genera *Thermosipho*, *Geotoga*, *Petrotoga*, *Thermotoga*, and *Pseudothermotoga* are widespread in oilfield-produced waters (Ollivier and Cayol, 2005; Dahle et al., 2008; Ollivier et al., 2014; Bhandari and Gupta, 2014; Vigneron et al., 2017). To date, the genus *Pseudothermotoga* contains seven species, *Pseudothermotoga elfii*, *Pseudothermotoga lettingae*, *Pseudothermotoga subterranea*, *Pseudothermotoga hypogea*, *Pseudothermotoga thermarum*, *Pseudothermotoga caldifontis*, and *Pseudothermotoga profunda*. However, *P. lettingae* and *P. subterranea* were recently proposed to be heterotypic synonyms of *P. elfii* based on comparative genomic analyses (Belahbib et al., 2018). Throughout the manuscript, we, therefore, use the proposed amended classification of these isolates. The *P. elfii* type strain, *P. elfii* strain DSM9442, has been isolated from a deep African oil-producing well (1600–1900 m depth) with an *in situ* temperature of 68°C (Ravot et al., 1995). Until now, all strains of *P. elfii* have originated from subsurface environments with the exception of *P. elfii* subsp. *lettingae* (DSM14385; Belahbib et al., 2018) which has been isolated from a thermophilic sulfate-reducing bioreactor operated at 65°C and at atmospheric pressure with methanol as the sole substrate (Balk et al., 2002). These two strains belonging to the same species give us the opportunity to better understand how microorganisms respond and adapt to hydrostatic pressure when they originate from either subsurface (*P. elfii* DSM9442) or surface (*P. elfii* subsp. *lettingae*) environments. Here, we report the growth characteristics of *P. elfii* DSM9442 and *P. elfii* subsp. *lettingae* under varying hydrostatic pressure conditions. To our knowledge, similar studies regarding the response to hydrostatic pressure involving two microorganisms pertaining to the same species but having different ecological niches (surface vs. subsurface) have never been documented.

## Materials and Methods

### Growth Medium and Bacterial Strains

The cultivation medium for cultures of both *P. elfii* DSM9442 and *P. elfii* subsp. *lettingae* (DSM14385) was based on the standard culture medium (Ravot et al., 1995) without sugar to avoid Maillard’s reaction, which can occur at high pressure and temperature. The modified medium contained, per liter of distilled water: thiosulfate 3 g, KH_2_PO_4_ 0.3 g, K_2_HPO_4_ 0.3 g, NH_4_Cl 1 g, CaCl_2_.2H_2_O 0.2 g, KCl 0.1 g, NaCl 10 g, MgCl_2_·6H_2_O 0.2 g, Cysteine-HCl 0.5 g, 0.1% resazurin 1 ml, Balch’s trace mineral solution 10 ml (Balch et al., 1979), and biotrypcase (Panreac, Spain) 2 g, as sole complex carbon and energy source. The pH was adjusted to 7 with KOH 10 M. The medium was boiled under a stream of O_2_-free N_2_ gas and cooled to room temperature. For routine growth at atmospheric pressure (0.1 MPa), Hungate tubes containing 5 ml of medium were prepared under a stream of N_2_-CO_2_ (80:20). Penicillin vials containing 40 ml of medium were dedicated to the common inoculum (at 0.1 MPa) for the kinetics experiments under various hydrostatic pressures. The tubes and vials were autoclaved for 20 min at 120°C. Prior to inoculation, 0.1 ml of 2% Na_2_S·9H_2_O, 0.1 ml of 10% NaHCO_3_, and 0.1 ml of Balch’s Vitamins (Balch et al., 1979), were added after autoclaving for 5 ml of medium. Cultures were inoculated at a 1:10 ratio and grown anaerobically at 65°C.

### Cultivation for Growth Kinetics

Cultures at high hydrostatic pressure were performed in 500-ml high-pressure bottles (HPBs, i.e., HPB-500). The hydrostatic pressure was controlled using a piloted pressure generator (PMHP 600-600, Top Industrie, Vaux-le-Pénil, France) connected to the HPBs (Tanet et al., 2019). For the growth kinetics, two consecutive subcultures of the *P. elfii* strains were grown from frozen stocks in fully filled 18-ml Hungate tubes at 65°C at the prescribed hydrostatic pressure for 63 h. Then, the last cultures were used to inoculate 5 ml of fresh medium in 10-ml gastight glass syringes (Socorex®, D. Dutscher, France) closed by a piece of rubber septum at the end of the needle. Triplicate cultures in gastight glass syringes were placed into the HPBs, pressurized at the prescribed hydrostatic pressure, and incubated at 65°C. To avoid decompression-recompression of the samples, each HPB corresponded to a specific incubation time (0, 6, 12, 16, 20, 36, or 63 h). For each incubation time, the content of each syringe was transferred to an empty Hungate tube under anaerobic conditions. Bacterial growth was monitored with cell counting using epifluorescence microscopy after the DAPI staining procedure. Briefly, 1 ml of each sample was fixed with 27 μl of 37% formaldehyde (Sigma-Aldrich, France) for 15 min at room temperature. Then, the samples were filtered on 0.22-μm pore-size polycarbonate filters. Filters were stained with DAPI, mounted on glass slides using nonfluorescent immersion oil and coverslips, and stored at −20°C until epifluorescence counting.

### End-Products Analysis

Culture samples (1,000 μl) were collected immediately after inoculation and after growth (63 h). Then, they were centrifuged for 10 min at 13,400 g (4°C). The supernatants were frozen in liquid nitrogen and stored at −20°C until use. Volatile FAs were quantified using a high-performance liquid chromatography (HPLC; UltiMate 3000, Thermo Scientific) equipped with an Aminex HPX-87H-300 × 7.8 mm column C18 (Bio-Rad). The column temperature was 35°C and eluant (H_2_SO_4_, 0.005 N) was used at a flow rate of 0.6 ml min^−1^. Twenty microliters of supernatant were injected. The thiosulfate concentration was measured on an ion chromatograph (761 Compact IC, Metrohm) equipped with an anion exchange column (Metrosep A Supp1 – 250/4.6). Na_2_CO_3_ (3 mM) was used as the mobile phase at a flow rate of 1 ml min^−1^ during the first 8 min, followed by a flow rate of 2.5 ml min^−1^. The culture supernatants were diluted 10-fold before injection (20 μl). Hydrogen sulfide was quantified spectrophotometrically according to the Cord-Ruwisch method (Cord-Ruwisch, 1985).

### Microscopy Analyses and Cell Counting

Microscopic observations were performed on an epifluorescence microscope (BX61 equipped with a 100-W-Hg-lamp and appropriate filter sets for DAPI, Olympus, France, and an 8-bit CCD camera C1140 ORCA flash 4.0, Hamamatsu). For cell counting, microscopic fields were photographed with the Olympus cellSens™ software (15–20 fields randomly per filter; Van Wambeke, 1988), and pictures were analyzed using CellC 12 software (Selinummi et al., 2005). The average cell count of each sample was estimated according to the following formula:

(1) $$B=\mathrm{Ncell}\times \frac{\mathrm{Sf}}{\mathrm{Sg}}\times \frac{1}{\mathrm{Vfi}}\times \frac{\mathrm{Vsamp}+\mathrm{Vformol}}{\mathrm{Vsamp}}\times \frac{1}{D}$$

where, B = the total number of cells per ml, Ncell = the average number of cells per field counted by CellC 12, Sf = the surface filtration – μm^2^ (for a 0.25 mm diameter filter, Sf = 2.6 × 10^8^ μm^2^), Sg = the grid surface – μm^2^ (for our microscope Sg = 133 μm × 133 μm), Vfi = volume of filtration – ml, Vsamp = the volume of sample – ml, Vform = the volume of formaldehyde 37% – ml, and D = the dilution of the sample. The growth rates were determined by fitting cell counts to the logistic model (Martini et al., 2013) using the web application https://hpteam.shinyapps.io/logistic_microbio/. The cell morphology was analyzed by transmission microscopy using ImageJ software (Eliceiri et al., 2012).

### Electron Microscopy

The samples were adsorbed on carbon-formvar copper grids (200 mesh), negatively stained with 1% uranyl acetate, and examined with a Philips MORGAGNI 268 electron microscope operating at 80 kV.

### Viability Assays

Viability assays were performed using the LIVE/DEAD™ FIXABLE RED DEAD Stain Cell Kit, 488 nm excitation (Invitrogen/Life Technologies, France). Cultures of *P. elfii* DSM9442 at various hydrostatic pressures were performed as described above. Ten-millimeter cultures in the exponential growth phase were transferred using an equi-pressure protocol as previously described (Garel et al., 2019) to a second HPB containing 10 μl of red fluorescent dye (diluted 1/100 in DMSO). The mix was incubated for 30 min at room temperature in the dark. The HPB was then slowly depressurized, and 270 μl of 37% formaldehyde was added to the mix and further incubated at room temperature for 15 min for fixation. Samples were then filtrated onto 0.22-μm pore-size polycarbonate filters, dried, and mounted on glass slides using nonfluorescent immersion oil and coverslips. The slides were stored at −20°C until use. Live and dead cells were visualized and photographed on an epifluorescence microscope equipped with a CY3 filter using Olympus cellSens™ software. Manual counting was performed from the analysis of at least 15 randomly selected fields. The viability rates were obtained from three biological replicates at 0.1 MPa and from one biological experiment at high pressure.

### Lipid Analysis

For lipid analysis, large-volume cultures were grown in Schott bottles (8 × 125 ml) that were fully filled. The bottles were inserted into several HPBs-500; the hydrostatic pressure applied to the HPBs was transmitted to the culture *via* the septum of the Schott bottle closed with a modified plastic screw cap (Duran group, Germany) including a rubber septum (Fisher Scientific, France). After incubation for 63 h at 65°C at the required hydrostatic pressure, the cells were collected by centrifugation (9,600 g for 20 min at 15°C). Cell pellets were lyophilized and stored at −20°C until further use. FA analysis was performed by the Identification Service of the DSMZ (Deutsche Sammlung von Mikroorganismen und Zellkulturen GmbH, Braunschweig, Germany; https://www.dsmz.de/).

### Statistics

Statistical analyses were performed with XLSTAT (Addinsoft) using the Mann-Whitney test, the Kruskal-Wallis test, or the *z*-test. Values were considered different at the *α* = 0.01 significance level.

## Results

### Growth Kinetics Experiments

Both *P. elfii* DSM9442 and *P. elfii* subsp. *lettingae* are able to use glucose as their energy source for growth (Ravot et al., 1995; Balk et al., 2002). However, Maillard’s reaction occurs under a high temperature and pressure yielding a dark yellow-brown coloration of the culture medium; therefore, glucose was omitted in all culture media, and biotrypcase was used as the sole complex carbon and energy source. Cultures of *P. elfii* DSM9442 at 0.1, 10, 20, 25, 30, 35, 40, and 50 MPa at 65°C revealed that this strain was able to grow up to 40 MPa, with no growth observed at 50 MPa after 5 days of incubation. Growth assays of *P. elfii* subsp. *lettingae* at 0.1, 20, and 30 MPa revealed that it was able to grow up to 20 MPa with no growth observed at 30 MPa after 5 days of incubation.

**Figure 1:**
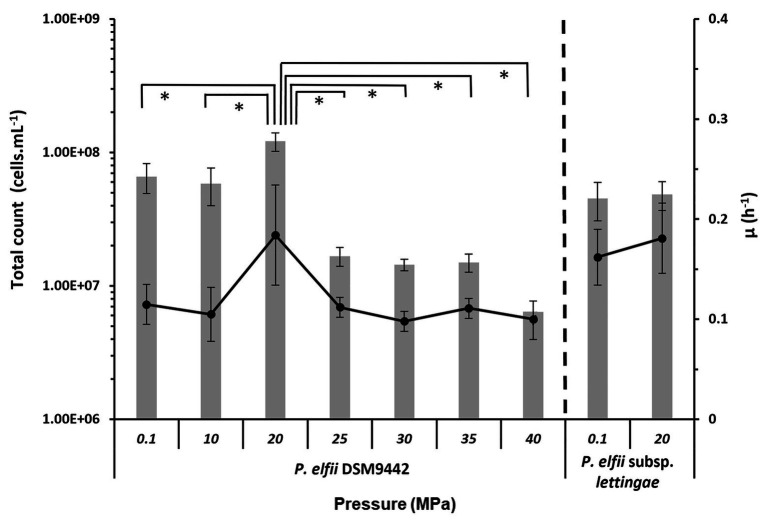
Final biomass and maximal growth rates achieved by the cultures of *Pseudothermotoga elfii* DSM9442 and *P. elfii* subsp. *lettingae* grown at various hydrostatic pressures. The final biomasses estimated from total cells count are represented as gray bars (left axis), and the maximal growth rates calculated from the growth curves are shown as in black lines (right axis). Data were obtained from at least three independent cultures. The values that are significantly different between the samples (*p* < 0.0001, *α* = 0.01, Mann-Whitney test) are marked with an asterisk.

The final biomass obtained from both strains cultured at various hydrostatic pressures was estimated from total cell counts (Figure 1). The maximal biomass for *P. elfii* DSM9442 was obtained when cells were grown at 20 MPa with up to 1.15 × 10^8^ cells ml^−1^ as the median value (0.9 × 10^8^ cells ml^−1^ minimum and 1.69 × 10^8^ cells ml^−1^ maximum). This maximal biomass was significantly higher (*p* < 0.001, Mann-Whitney test) at 20 MPa than that measured at the other pressures. Concerning *P. elfii* subsp. *lettingae*, the final biomasses obtained when cultures were grown at 0.1 and 20 MPa were similar (*p* > 0.05, Mann-Whitney test), with median values of 4.47 and 4.68 × 10^7^ cells ml^−1^, respectively.

The maximal growth rates deduced from the growth kinetics curves obtained at various hydrostatic pressures are also presented in Figure 1. Growth curves of *P. elfii* DSM9442 and *P. elfii* subsp. *lettingae* cultured at various hydrostatic pressures are shown in Supplementary Figure 1. The maximal growth rate for *P. elfii* DSM9442 was higher at 20 MPa (0.184 h^−1^) than at the other hydrostatic pressures (<0.114 h^−1^), whereas it was quite similar for *P. elfii* subsp. *lettingae* grown at either 0.1 or 20 MPa (0.162 and 0.181 h^−1^, respectively).

### End-Products Analysis

**Figure 2:**
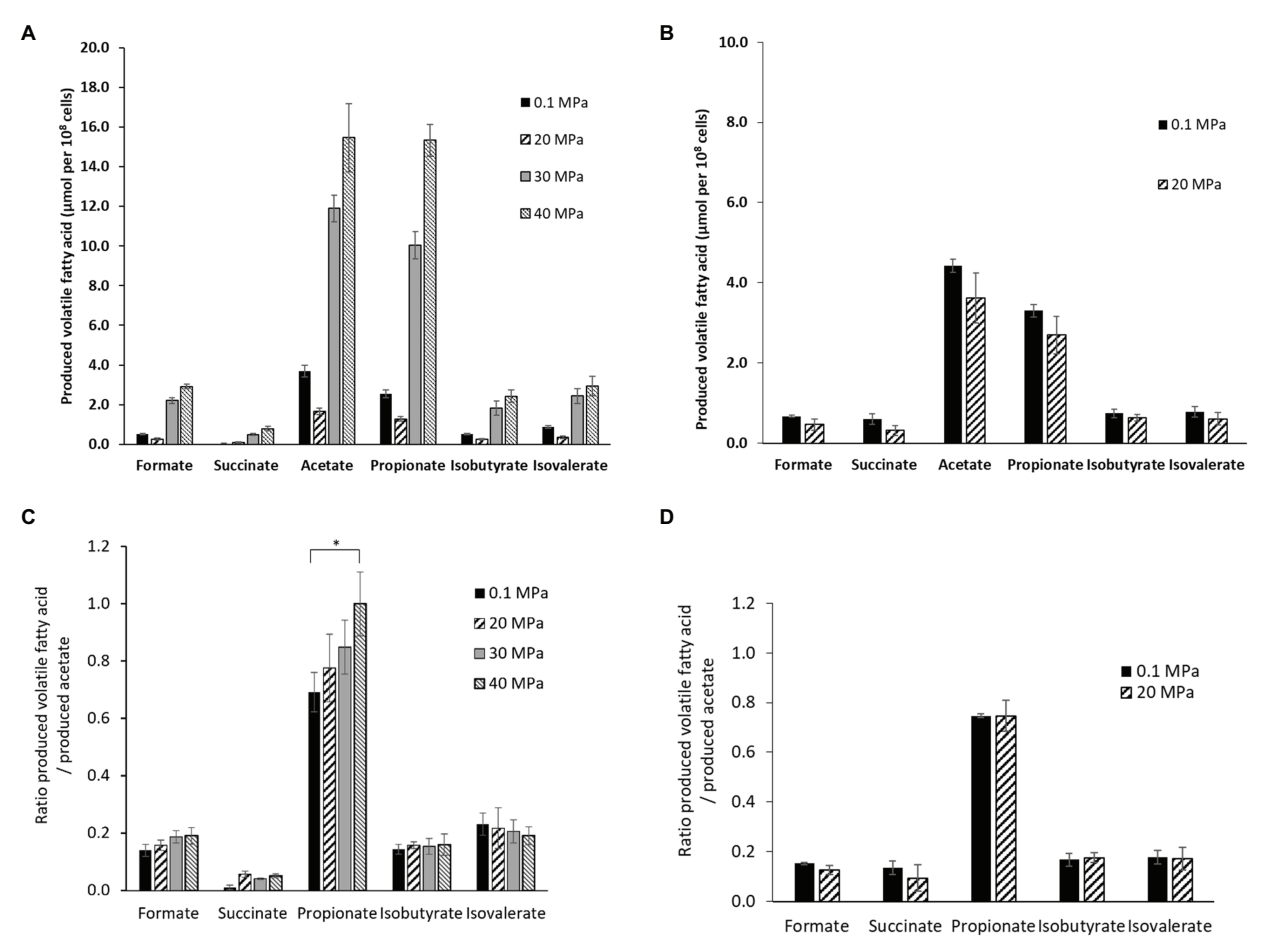
Volatile fatty acids (FAs) production by *P. elfii* DSM9442 **(A)**, and *P. elfii* subsp. *lettingae* **(B)**, expressed as μmol per 10^8^ cells and ratios of produced volatile FAs related to the amount of acetate produced by *P. elfii* DSM9442 **(C)**, and *P. elfii* subsp. *lettingae* **(D)** when grown at various hydrostatic pressures. Significant increase in the produced-propionate to produced acetate ratio between 0.1 and 40 MPa is marked with one asterisk (*p* = 0.003, *α* = 0.01, Kruskal-Wallis test).

The end-products of the metabolism were analyzed by HPLC on culture supernatants of *P. elfii* DSM9442 grown at 0.1, 20, 30, and 40 MPa and of *P. elfii* subsp. *lettingae* grown at 0.1 and 20 MPa. Fermentation of either peptides or amino acids contained in the biotrypcase led to the production of acetate and propionate by *P. elfii* DSM9442 and *P. elfii* subsp. *lettingae*. Formate, succinate, isobutyrate, and isovalerate were produced to lower extents, regardless of the hydrostatic pressure applied to the cultures (Figure 2). Figure 2A shows that, for the same produced biomass, a lower amount of volatile FAs was obviously produced by *P. elfii* DSM9442 grown at 20 MPa than at the other hydrostatic pressures. This finding suggested that, at 20 MPa, the metabolism of *P. elfii* DSM9442 was optimal. This phenomenon was not observed for *P. elfii* subsp. *lettingae*, where the production of volatile FAs for the same biomass did not vary distinctly (Figure 2B). Overall, the production levels of the various volatile FAs compared to acetate were not manifestly affected by the hydrostatic pressure for either strain (Figures 2C,D), except for the produced-propionate to the produced-acetate ratio for *P. elfii* DSM9442, which slightly increased with the increase in the hydrostatic pressure (Figure 2C). These findings suggest that the propionate production pathway was stimulated at elevated hydrostatic pressures.

**Figure 3:**
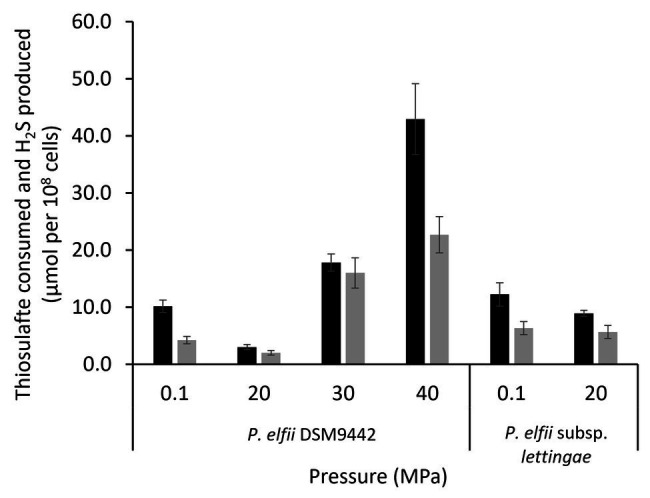
Thiosulfate consumption (black bars) and H_2_S production (gray bars) by *P. elfii* DSM9442 and *P. elfii* subsp. *lettingae* (expressed as μmol per 10^8^ cells) grown at various hydrostatic pressures.

It has been previously shown that the presence of thiosulfate enhanced the growth of both *P. elfii* DSM9442 and *P. elfii* subsp. *lettingae*, and both strains produced hydrogen sulfide under this condition (Ravot et al., 1995; Balk et al., 2002). Thiosulfate can also be used as a sulfur source for the synthesis of cellular materials, as shown for *Thermotoga maritima* (Boileau et al., 2016). Consumption of thiosulfate was thus measured in the cultures grown at the various hydrostatic pressures. Figure 3 shows that consumption of thiosulfate and production of H_2_S was quite similar for the *P. elfii* subsp. *lettingae* cultures grown at 0.1 and 20 MPa, respectively. In contrast, thiosulfate consumption decreased when *P. elfii* DSM9442 was grown at 20 MPa compared to other pressures (Figure 3). The highest thiosulfate consumption was measured when *P. elfii* DSM9442 was grown at 40 MPa. Consequently, H_2_S production was the lowest when cells were grown at 20 MPa and the highest when cells were grown at 40 MPa. Moreover, the consumed-thiosulfate to produced-acetate ratio was lower when *P. elfii* DSM9442 was grown at 20 MPa (1.81 ± 0.22) than at 0.1 or 40 MPa (2.76 ± 0.23 or 2.83 ± 0.42, respectively), indicating a reduced need to release excess reducing power at 20 MPa.

### Cell Morphology According to Pressure

The effects of hydrostatic pressure on the morphology of the two bacteria were analyzed by microscopy. Both *P. elfii* DSM9442 and *P. elfii* subsp. *lettingae* have been defined as rod-shaped organisms of 0.5–1 by 2–3 μm in size, occurring singly or in pairs, and surrounded by a characteristic toga, a sheath-like structure ballooning over the ends (Ravot et al., 1995; Balk et al., 2002; Supplementary Figure 2). The analysis of the morphology of cells in the early stationary phase by transmission microscopy revealed that the length of *P. elfii* DSM9442 single cells was not significantly affected by the pressure (*p* > 0.01, Mann-Whitney test), with median values ranging from 2.43 to 2.74 μm. The same observation was found for the paired cells; however, when the cells were grown at 40 MPa, the length of the paired cells significantly increased (*p* < 0.0001, Mann-Whitney test) compared to cells grown at 0.1 MPa, 5.20 vs. 4.52 μm, respectively (Supplementary Figure 3). *Pseudothermotoga elfii* subsp. *lettingae* paired cells appeared slightly longer at 20 than at 0.1 MPa (4.03 vs. 4.76 μm; *p* < 0.0001, Mann-Whitney test), suggesting that hydrostatic pressure affected the length of the paired cells (Supplementary Figure 3). No significant difference in the length of the single cells was observed (*p* > 0.01, Mann-Whitney test).

**Figure 4:**
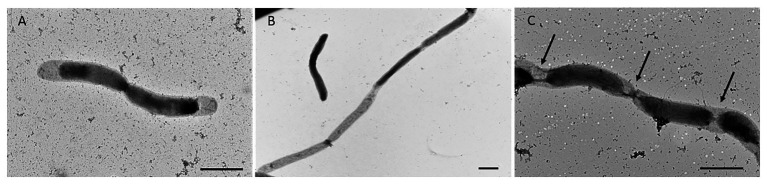
Electron microscopy (TEM) photographs of *P. elfii* DSM9442 grown at 40 MPa in the early stationary growth phase. Paired cells **(A)** and chained cells **(B,C)**. The arrows indicate the location of the septa between cells in the chains. Scale bar = 1 μm.

**Figure 5:**
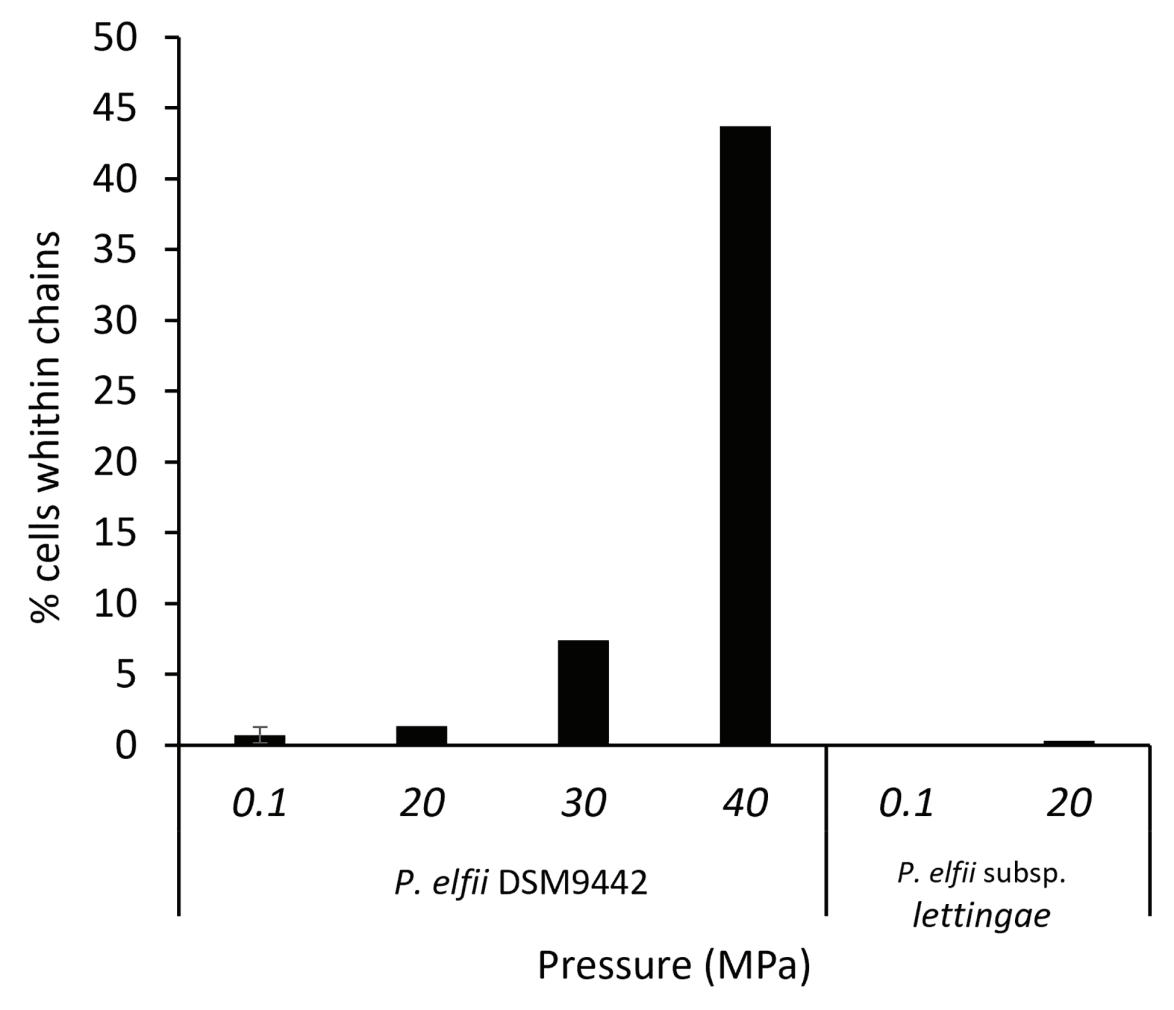
Percentages of chained cells in early stationary growth phase cultures of *P. elfii* DSM9442 and *P. elfii* subsp. *lettingae* grown at various hydrostatic pressures.

**Figure 6:**
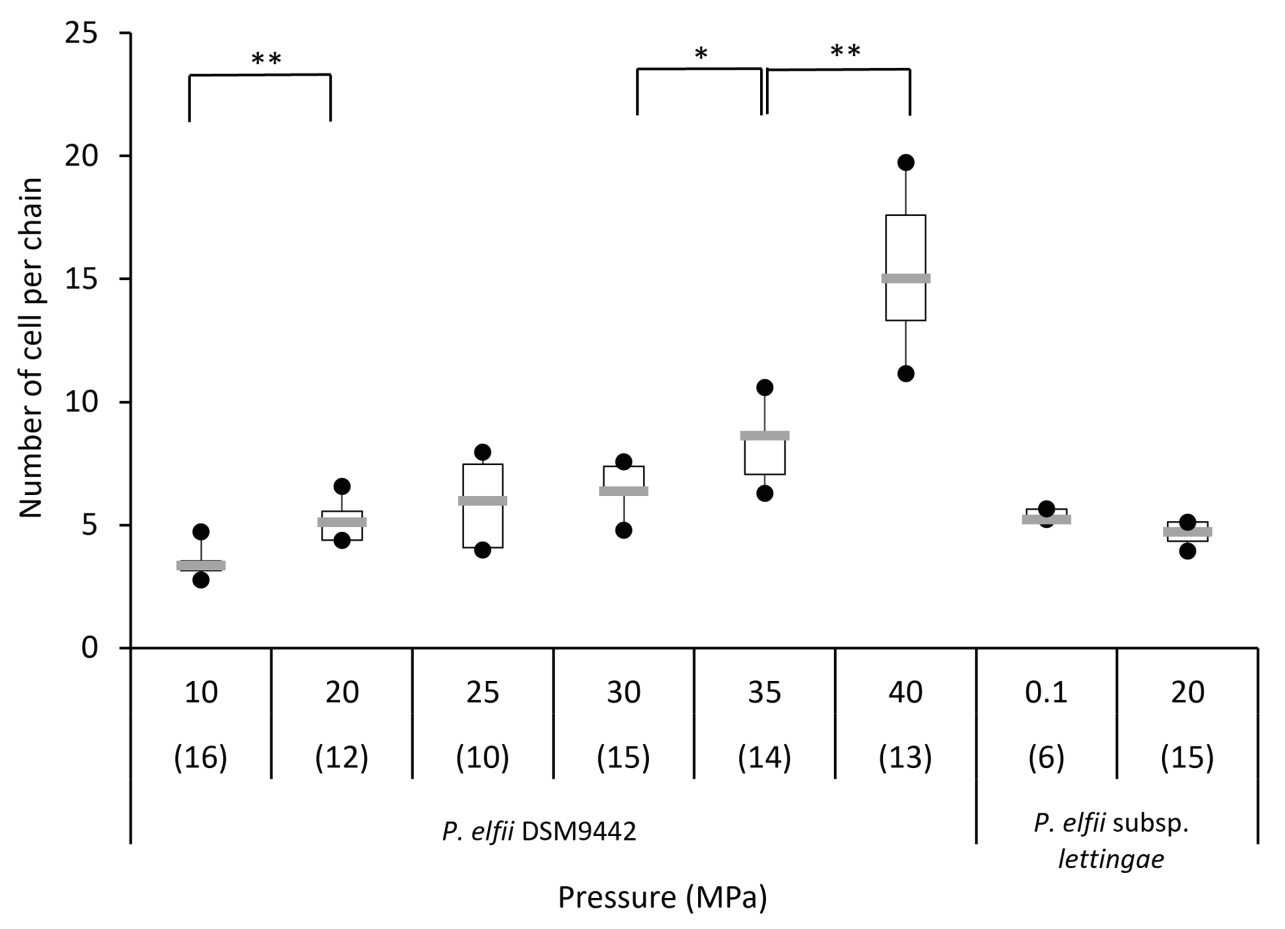
Distribution of the number of cells per chain of *P. elfii* DSM9442 and *P. elfii* subsp. *lettingae* in the early stationary growth phase at various hydrostatic pressures represented as box and whiskers plots. The number in brackets corresponds to the number of analyzed chains for each pressure. Significant increases in the number of cells per chain with increasing hydrostatic pressure are marked with one (*p* < 0.01) and two (*p* < 0.0001) asterisks (Mann-Whitney test, *α* = 0.01).

In addition to single and paired cells, a third morphotype was also observed in both *P. elfii* DSM9442 and *P. elfii* subsp. *lettingae* cultures composed of chains including more than two cells (Supplementary Figure 4). Electron microscopy (TEM) photographs show that cells included in the chain are individualized (Figure 4). In the case of *P. elfii* DSM9442, the number of chained cells increased from 0.07% of the total cells at 0.1 MPa to 44% at 40 MPa; in the case of *P. elfii* subsp. *lettingae*, the number of chained cells was higher at 20 than at 0.1 MPa but always remained low (0.3% at 20 MPa; Figure 5). It should be noted that the proportion of chained cells was quite similar regardless of the time of growth (Supplementary Figure 5), indicating that chain formation was independent of the growth phase and instead was a hydrostatic pressure dependent-phenomenon. In addition, the sizes of the cells within the chains were similar to the sizes of single cells (data not shown). Figure 6 shows that the average number of cells per chain increased with the increase in hydrostatic pressure to reach ~15 cells per chain when *P. elfii* DSM9442 was cultured at 40 MPa. In contrast, the number of cells per chain for *P. elfii* subsp. *lettingae* was ~5 when grown at both 20 and 0.1 MPa (Figure 6).

### Cell Viability at Various Hydrostatic Pressures

**Figure 7:**
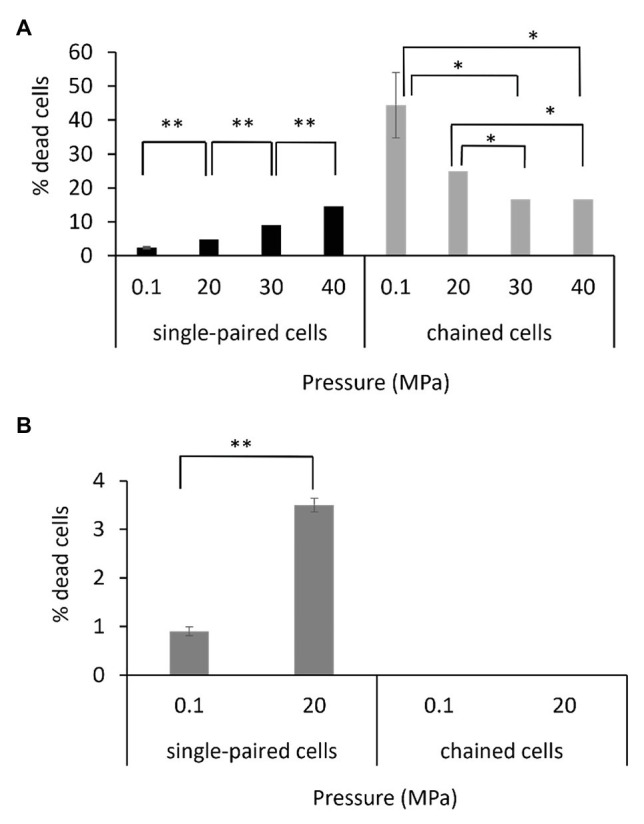
Viabilities of single-paired cells and chained cells of *P. elfii* DSM9442 **(A)** and *P. elfii* subsp. *lettingae* **(B)** collected during the exponential phase at various hydrostatic pressures, expressed as percentages of dead cells within each morphotype. One asterisk (*) indicates a significant difference between samples with *p* < 0.01 (*z*-test, *α* = 0.01) and two asterisks (**) indicate *p* < 0.001 (*z*-test, *α* = 0.01).

The viability of the single-paired cells and of the chained cells was assessed by using a fluorescent dye that binds only to the periphery of the cell when membrane integrity is maintained (live cell), resulting in a low level of fluorescence, whereas it penetrates into the cell when the membrane is altered (dead cell), resulting in a higher level of fluorescence (Supplementary Figure 6). The protocol was set up to perform the staining reaction under pressure to limit the bias that could have been introduced if the fluorescent dye was added after a depressurization step. Assays were performed on exponentially growing cells. Interestingly, the percentage of dead cells as a function of hydrostatic pressure differed for the *P. elfii* DSM9442 morphotypes (i.e., single-paired cells and chained cells). The percentage of dead single-paired cells increased significantly (*p* < 0.001, *z*-test) with increasing hydrostatic pressure; in contrast, the percentage of dead chained cells was significantly lower at 30 and 40 MPa than at 0.1 and 20 MPa (*p* < 0.01, *z*-test; Figure 7A). These findings suggest a survival benefit for cells in chains when they are grown at elevated hydrostatic pressures. It should be noted that all cells constituting a chain were in the same state, either dead or alive (data not shown). In the case of *P. elfii* subsp. *lettingae*, the percentage of dead single-paired cells was also higher at 20 MPa compared to 0.1 MPa (*p* < 0.001, *z*-test), whereas no dead cells were observed within chains at either hydrostatic pressure (Figure 7B); however, the overall number of chains was very low regardless of hydrostatic pressure.

### Pressure Effects on the Cellular Fatty-Acid Composition

The FA compositions were similar for both *P. elfii* DSM9442 and *P. elfii* subsp. *lettingae*, with the major FAs being C_16:0_ (>45%), C_18:1w9c_ (12.71–18.95%), C_18:0_ (11.44–19.99%), and C_18:2 w6,9c_ (5.87–9.08%; Table 1). While no clear correlation was found between the changes in the percentages of the longest (C_18_ + C_20_) and total unsaturated FAs and hydrostatic pressures, several observations should be noted: decreases in the amounts of saturated C14 for both *P. elfii* DSM9442 and *P. elfii* subsp. *lettingae* with increasing pressure and an increase in the total branched iso-anteiso and the detection of unsaturated C_20:2 w6,9c_ and C_18:1 w5c_ at elevated hydrostatic pressures compared to atmospheric pressure (Table 1). These observations suggest a fine modification of the membrane composition to manage elevated hydrostatic pressures.

**Table 1:** Fatty acids composition of the two strains grown at 65°C under different hydrostatic pressure conditions (values are expressed in %).

	*P. elfii* subsp. *lettingae*		*P. elfii* DSM9442			
Fatty acid type	0.1 MPa	20 MPa	0.1 MPa	20 MPa	30 MPa	40 MPa
C_14:0_	1.7	1.06	2.48	2.22	1.06	1.06
C_15:0_	0.51	0.93	1.18	0.78	0.86	0.59
C_16:0_	47.14	45.2	50.1	53.37	53.49	45.71
C_16:0 iso_	0.75	1.32	0.78	1.46	1.15	1.47
C_16:1 w7c_		1.21				
C_17:0 anteiso_	0.47	0.63	0.87	0.75	0.8	0.68
C_17:0_	1.69	2.86	3.22	2.98	3.64	3.18
C_18:1 w9c_	17.53	14.54	18.18	15.64	12.71	18.95
C_18:1 w7c_	2.38	2.35	2.66	2.2	1.86	2.75
C_18:1 w5c_						0.44
C_18:0_	18.72	19.99	11.44	13.95	18.13	15.78
C_18:2 w6,9c_	6.69	7.84	9.08	6.65	5.87	8.43
C_20:2 w6,9c_	1.12	1.2			0.44	0.95
C_20:1 w9c_	0.6					
C_20:0_	0.7	0.86				
Total longest (C_18_ + C_20_)	47.74	46.78	41.36	38.44	39.01	47.3
Total unsaturated (:1 + :2)	28.32	27.14	29.92	24.49	20.88	31.52
Total branched iso-anteiso	1.22	1.95	1.65	2.21	1.95	2.15

## Discussion

In this study, the differing behaviors of two *P. elfii* strains, originating from either subsurface (*P. elfii* DSM9442) or surface (*P. elfii* subsp. *lettingae*) environments, with respect to hydrostatic pressure, has been highlighted. The final biomass and growth rate indicate that the growth of *P. elfii* DSM9442, isolated from a deep-oil reservoir, is better adapted to high hydrostatic pressure than to atmospheric pressure, with an optimal hydrostatic pressure of 20 MPa (tolerance up to 40 MPa). In contrast, the growth attributes of *P. elfii* subsp. *lettingae*, isolated from a bioreactor at atmospheric pressure, are quite similar at atmospheric pressure and at 20 MPa. Additionally, the pressure tolerance range of *P. elfii* subsp. *lettingae* is narrow, as no growth occurred above 30 MPa. With respect to these findings, *P. elfii* DSM9442 should be recognized as a piezophilic microorganism with an optimal hydrostatic pressure of 20 MPa, which corresponds to the depth of the oil reservoir (1,600–1,900 m) from which this bacterium has been isolated. In contrast, *P. elfii* subsp. *lettingae* is merely piezotolerant.

In the experiments described here, the only sources of carbon and energy are peptides and/or amino acids. Energy conservation arises from amino acid fermentation which leads to the formation of ATP by substrate-level phosphorylation. The catabolic pathway of amino acids generates ammonia, which can be used as a nitrogen source, as well as short-chain FAs, including acetate, propionate, formate, succinate, and branched-chain FAs, including isovalerate and isobutyrate (Barker, 1981). *Pseudothermotoga elfii* DSM9442 and *P. elfii* subsp. *lettingae* produce acetate and propionate as major volatile FAs, whereas isovalerate, formate, succinate, and isobutyrate were produced to lower extents. It can therefore be assumed that both strains would metabolize preferential amino acids yielding mostly the short-chain FAs acetate and propionate and, to a lower extent, branched-chain amino acids that lead to the production of the branched-chain FAs isovalerate and isobutyrate. The favored use of specific amino acids by a microorganism has already been reported (Rees et al., 1998). No significant variation in volatile FA production has been observed with respect to the hydrostatic pressure, except for propionate production, which increases slightly with increasing hydrostatic pressure in *P. elfii* DSM9442 cultures. This suggests that hydrostatic pressure stimulates fermentation routes for propionate production in this strain, such as the acrylate or succinate pathways, and/or the catabolic pathways of some specific amino acids (threonine, methionine, isoleucine, and valine) that lead to the production of propionate and ATP *via* propionyl-CoA (Gonzalez-Garcia et al., 2017). In addition to substrate-level phosphorylation, energy conservation *via* electron transport phosphorylation can also be achieved by the Rnf complex (Biegel et al., 2011; Fadhlaoui et al., 2018). This complex can catalyze the electron flow from reduced ferredoxin, generated by amino acid fermentation pathways, to NAD+, coupled with ion translocation across the membrane, thus allowing chemiosmotic energy generation. While the presence of thiosulfate enhances the growth of both *P. elfii* strains, its reduction to hydrogen sulfide is not considered an energy-producing respiratory process but rather a detoxifying process through elimination of excessive reducing power (Ravot et al., 1995; Balk et al., 2002). Thiosulfate consumption, hydrogen sulfide production, and the consumed-thiosulfate to the produced-acetate ratio are the lowest when *P. elfii* DSM9442 is cultured at 20 MPa, suggesting that there is no need to eliminate excess reducing power, indicating that the coupling between catabolism and anabolism is optimal at this hydrostatic pressure, which is an additional confirmation of the piezophilic nature of *P. elfii* DSM9492.

Increases in unsaturated FAs and/or in FA chains length have been reported to be involved in hydrostatic pressure adaptation in several bacteria (Allen et al., 1999; Allen and Bartlett, 2002; Bartlett, 2002; Simonato et al., 2006; Abe, 2013; Pradel et al., 2013). In this study, we found that even if the global profiles of the FA compositions were similar, increases in the branched iso-anteiso FAs and in unsaturated long-chain C_20:2 w6,9c_ and C_18:1 w5c_ were observed at elevated hydrostatic pressures compared to atmospheric pressure for both *P. elfii* strains. This suggests a fine modification of the membrane composition to manage the FA ratio and thus maintain the membrane homeoviscosity at high pressure. Indeed, increases in the levels of lower-melting-temperature branched FAs in piezophilic bacteria have been reported to increase the membrane fluidity (DeLong and Yayanos, 1985; Kates, 1986; Yano et al., 1998). We can hypothesize that this modification may be enough to increase the fluidity of the membranes under high pressure for both *P. elfii* strains. Such particularities have never been reported for the *Thermotogales*.

The adaptations to high hydrostatic pressure at the morphological level differ between the two *P. elfii* strains: while the hydrostatic pressure does not influence the length of *P. elfii* DSM9442 cells up to 35 MPa, it induces a significant increase in the *P. elfii* subsp. *lettingae* paired cells size (+25% cell length) at 20 MPa. Both *Pseudothermotoga* strains form chained cells when the hydrostatic pressure increases; however, whereas *P. elfii* subsp. *lettingae* chained cells are rare, they are numerous for *P. elfii* DSM9442, as they account for 44% of the cells at the highest hydrostatic pressure tolerated (40 MPa). In addition, the number of *P. elfii* DSM9442 cells per chain increases with the increase in the hydrostatic pressure, reaching up to 15 cells per chain at 40 MPa. It is noteworthy that chain formation in *P. elfii* DSM9442 occurs mainly at 40 MPa and is quite rare at 20 MPa, which is the optimal hydrostatic pressure for this strain. The chained morphotype has not been previously described in *Pseudothermotoga* species. It has been reported that when a microorganism is grown above its optimal hydrostatic pressure, it tends to become filamentous (Yayanos and Delong, 1987). This phenomenon has been proposed to be related to a septation default involving FtsZ ring formation (Aertsen et al., 2004; Ishii et al., 2004). In this study, TEM images of the pressure-induced chains clearly show separated cells within chains, indicating that septation of chained cells is not affected by the hydrostatic pressure. This observation suggests that chain formation is related to a modification of the toga structure rather than a default of cell septation. However, further analyses are needed, notably at the genomic and transcriptomic levels, to determine the biological mechanisms, regulatory elements, and key genes that govern chain formation. In addition, the death rate of *P. elfii* DSM9442 chained cells is lower at high hydrostatic pressure (40 MPa) than at atmospheric pressure, and the number of dead single or paired cells increases with the increase in the hydrostatic pressure for both *P. elfii* strains. This indicates that chain formation in *Pseudothermotoga* species should be considered as a protective mechanism that takes place when high hydrostatic pressure becomes deleterious rather than a degenerative phenomenon. Multicell chain formation has already been reported in members of the order *Thermotogales*. *Marinitoga piezophila* cells, which have been isolated from a deep-sea hydrothermal vent, appear singly or in chains within the sheath when cultivated at 40 MPa, the optimal pressure for growth. Under unfavorable conditions (lower hydrostatic pressure), cells appear elongated and twisted and chains with up to 10 cells can be occasionally observed (Alain et al., 2002). In *Thermosipho japonicus*, the formation of multicell chains has been reported depending on the growth phase, with the chain morphotype being more abundant in the stationary phase than in the early exponential phase. However, it should be noted that the experiments were conducted only at atmospheric pressure (Takai and Horikoshi, 2000). This contrasts with *P. elfii* DSM9442 in which chain formation does not depend on the growth phase. In addition, no twisted cells were observed when the strain was cultured under unfavorable conditions (at lower or higher hydrostatic pressure than the optimal one). In chains, the bacteria collectively possess a larger surface area. The advantage of chain formation has been mostly studied for pathogenic bacteria and has shown that chains confer adhesive properties and/or resistance advantage to the cells (Yang et al., 2016). In *P. elfii* DSM9442, one can hypothesize that chains favor intercell communication and exchanges and, thus, could confer to the cells an energy-saving advantage under high-pressure conditions. The less pronounced ability of *P. elfii* subsp. *lettingae* to form chains could explain its difficulties to adapt to high hydrostatic pressures. Overall, this morphological phenomenon highlights the different behavior of the two closely related strains with respect to hydrostatic pressure.

To our knowledge, this is the first time that a thermophilic anaerobic microorganism originated from a deep-oil reservoir has been described as piezophilic, thus suggesting that many other oil reservoir microbes, and particularly thermophilic anaerobes inhabiting deep-oil reservoirs, may share this physiological feature with *P. elfii* DSM9442. These findings also suggest that *P. elfii* DSM9442 is an autochthonous inhabitant of oil reservoirs, as it is well adapted to the *in situ* conditions rather than an anthropogenically introduced organism. In this respect, hydrostatic/lithostatic pressure can be expected to have a significant impact on the geomicrobiological cycles in these extreme environments and should therefore merit further investigation when considering subsurface life and global biogeochemical cycling of nutrients.

## Data Availability Statement

The original contributions presented in the study are included in the article/Supplementary Material; further inquiries can be directed to the corresponding authors.

## Author Contributions

ZS, BO, CT, FA, and AD contributed to conception and design of the study. MR, NP, MB, MG, AJ, and RF performed the experiments. All authors contributed to the article and approved the submitted version.

### Conflict of Interest

ZS and AJ were employed by the ExxonMobil Research and Engineering Company.

The remaining authors declare that the research was conducted in the absence of any commercial or financial relationships that could be construed as potential conflicts of interest.

## References

[ref1] Abe F. (2013). Dynamic structural changes in microbial membranes in response to high hydrostatic pressure analyzed using time-resolved fluorescence anisotropy measurement. Biophys. Chem. 183, 3–8. 10.1016/j.bpc.2013.05.005, PMID: 23790318

[ref2] Aertsen A. Van Houdt R. Vanoirbeek K. Michiels C. W. (2004). An SOS response induced by high pressure in *Escherichia coli*. J. Bacteriol. 186, 6133–6141. 10.1128/JB.186.18.6133-6141.2004, PMID: 15342583 PMC515162

[ref3] Alain K. Marteinsson V. T. Miroshnichenko M. L. Bonch-Osmolovskaya E. A. Prieur D. Birrien J. L. (2002). Marinitoga piezophila sp. nov., a rod-shaped, thermo-piezophilic bacterium isolated under high hydrostatic pressure from a deep-sea hydrothermal vent. Int. J. Syst. Evol. Microbiol. 52, 1331–1339. 10.1099/00207713-52-4-1331, PMID: 12148648

[ref4] Allen E. E. Bartlett D. H. (2002). Structure and regulation of the omega-3 polyunsaturated fatty acid synthase genes from the deep-sea bacterium *Photobacterium profundum* strain SS9. Microbiology 148, 1903–1913. 10.1099/00221287-148-6-1903, PMID: 12055309

[ref5] Allen E. E. Facciotti D. Bartlett D. H. (1999). Monounsaturated but not polyunsaturated fatty acids are required for growth of the deep-sea bacterium *Photobacterium profundum* SS9 at high pressure and low temperature. Appl. Environ. Microbiol. 65, 1710–1720. 10.1128/AEM.65.4.1710-1720.1999, PMID: 10103272 PMC91242

[ref6] Amrani A. Bergon A. Holota H. Tamburini C. Garel M. Ollivier B.. (2014). Transcriptomics reveal several gene expression patterns in the piezophile *Desulfovibrio hydrothermalis* in response to hydrostatic pressure. PLoS One 9:e106831. 10.1371/journal.pone.0106831, PMID: 25215865 PMC4162548

[ref7] Amrani A. van Helden J. Bergon A. Aouane A. Ben Hania W. Tamburini C.. (2016). Deciphering the adaptation strategies of *Desulfovibrio piezophilus* to hydrostatic pressure through metabolic and transcriptional analyses. Environ. Microbiol. Rep. 8, 520–526. 10.1111/1758-2229.12427, PMID: 27264199

[ref8] Balch W. E. Fox G. E. Magrum L. J. Woese C. R. Wolfe R. S. (1979). Methanogens: reevaluation of a unique biological group. Microbiol. Rev. 43, 260–296. PMID: 390357 10.1128/mr.43.2.260-296.1979 PMC281474

[ref9] Balk M. Weijma J. Stams A. J. (2002). *Thermotoga lettingae* sp. nov., a novel thermophilic, methanol-degrading bacterium isolated from a thermophilic anaerobic reactor. Int. J. Syst. Evol. Microbiol. 52, 1361–1368. 10.1099/00207713-52-4-1361, PMID: 12148651

[ref10] Barker H. A. (1981). Amino acid degradation by anaerobic bacteria. Annu. Rev. Biochem. 50, 23–40. 10.1146/annurev.bi.50.070181.000323, PMID: 6791576

[ref11] Bartlett D. H. (2002). Pressure effects on in vivo microbial processes. Biochim. Biophys. Acta 1595, 367–381. 10.1016/s0167-4838(01)00357-0, PMID: 11983409

[ref12] Belahbib H. Summers Z. M. Fardeau M. L. Joseph M. Tamburini C. Dolla A.. (2018). Towards a congruent reclassification and nomenclature of the thermophilic species of the genus *Pseudothermotoga* within the order *Thermotogales*. Syst. Appl. Microbiol. 41, 555–563. 10.1016/j.syapm.2018.04.007, PMID: 29801938

[ref13] Bhandari V. Gupta R. S. (2014). Molecular signatures for the phylum (class) Thermotogae and a proposal for its division into three orders (Thermotogales, Kosmotogales ord. nov. and Petrotogales ord. nov.) containing four families (Thermotogaceae, Fervidobacteriaceae fam. nov., Kosmotogaceae fam. nov. and Petrotogaceae fam. nov.) and a new genus *Pseudothermotoga* gen. nov. with five new combinations. Antonie Van Leeuwenhoek 105, 143–168. 10.1007/s10482-013-0062-7, PMID: 24166034

[ref14] Biegel E. Schmidt S. Gonzalez J. M. Muller V. (2011). Biochemistry, evolution and physiological function of the Rnf complex, a novel ion-motive electron transport complex in prokaryotes. Cell. Mol. Life Sci. 68, 613–634. 10.1007/s00018-010-0555-8, PMID: 21072677 PMC11115008

[ref15] Boileau C. Auria R. Davidson S. Casalot L. Christen P. Liebgott P. P.. (2016). Hydrogen production by the hyperthermophilic bacterium *Thermotoga maritima* part I: effects of sulfured nutriments, with thiosulfate as model, on hydrogen production and growth. Biotechnol. Biofuels 9:269. 10.1186/s13068-016-0678-8, PMID: 28018486 PMC5168592

[ref16] Cai M. Nie Y. Chi C. Q. Tang Y. Q. Li Y. Wang X. B.. (2015). Crude oil as a microbial seed bank with unexpected functional potentials. Sci. Rep. 5:16057. 10.1038/srep16057, PMID: 26525361 PMC4630613

[ref17] Cario A. Gina O. Rogers K. (2019). Exploring the deep marine biosphere: challenges, innovations, and opportunities earth science. Front. Earth Sci. 7:225. 10.3389/feart.2019.00225

[ref18] Cario A. Jebbar M. Thiel A. Kervarec N. Oger P. M. (2016). Molecular chaperone accumulation as a function of stress evidences adaptation to high hydrostatic pressure in the piezophilic archaeon *Thermococcus barophilus*. Sci. Rep. 6:29483. 10.1038/srep29483, PMID: 27378270 PMC4932500

[ref19] Cord-Ruwisch R. (1985). A quick method for the determination of dissolved and precipitated sulfides in cultures of sulfate-reducing bacteria. J. Microbiol. Methods 4, 33–36. 10.1016/0167-7012(85)90005-3

[ref20] Dahle H. Garshol F. Madsen M. Birkeland N. K. (2008). Microbial community structure analysis of produced water from a high-temperature North Sea oil-field. Antonie Van Leeuwenhoek 93, 37–49. 10.1007/s10482-007-9177-z, PMID: 17588160

[ref21] DeLong E. Yayanos A. (1985). Adaptation of the membrane lipids of a deep-sea bacterium to changes in hydrostatic pressure. Science 228, 1101–1103. 10.1126/science.3992247, PMID: 3992247

[ref22] Eliceiri K. W. Berthold M. R. Goldberg I. G. Ibáñez L. Manjunath B. S. Martone M. E.. (2012). Biological imaging software tools. Nat. Methods 9, 697–710. 10.1038/nmeth.2084, PMID: 22743775 PMC3659807

[ref23] Fadhlaoui K. Ben Hania W. Armougom F. Bartoli M. Fardeau M. L. Erauso G.. (2018). Obligate sugar oxidation in Mesotoga spp., phylum Thermotogae, in the presence of either elemental sulfur or hydrogenotrophic sulfate-reducers as electron acceptor. Environ. Microbiol. 20, 281–292. 10.1111/1462-2920.13995, PMID: 29124868

[ref24] Garel M. Bonin P. Martini S. Guasco S. Roumagnac M. Bhairy N.. (2019). Pressure-retaining sampler and high-pressure systems to study deep-sea microbes under in situ conditions. Front. Microbiol. 10:453. 10.3389/fmicb.2019.00453, PMID: 31024462 PMC6465632

[ref25] Gonzalez-Garcia R. A. McCubbin T. Navone L. Stowers C. Nielsen L. K. Marcellin E. (2017). Microbial propionic acid production. Fermentation 3:21. 10.3390/fermentation3020021

[ref26] Grossi V. Yakimov M. Al Ali B. Tapilatu Y. Cuny P. Goutx M.. (2010). Hydrostatic pressure affects membrane and storage lipid compositions of the piezotolerant hydrocarbon-degrading Marinobacter hydrocarbonoclasticus strain #5. Environ. Microbiol. 12, 2020–2033. 10.1111/j.1462-2920.2010.02213.x, PMID: 20406283

[ref27] Ishii A. Sato T. Wachi M. Nagai K. Kato C. (2004). Effects of high hydrostatic pressure on bacterial cytoskeleton FtsZ polymers in vivo and in vitro. Microbiology 150, 1965–1972. 10.1099/mic.0.26962-0, PMID: 15184582

[ref28] Jebbar M. Franzetti B. Girard E. Oger P. (2015). Microbial diversity and adaptation to high hydrostatic pressure in deep-sea hydrothermal vents prokaryotes. Extremophiles 19, 721–740. 10.1007/s00792-015-0760-3, PMID: 26101015

[ref29] Kates M. (1986). Influence of salt concentration on the membrane lipids of halophilic bacteria. FEMS Microbiol. Rev. 39, 95–101. 10.1016/0378-1097(86)90066-2

[ref30] Kotlar H. K. Lewin A. Johansen J. Throne-Holst M. Haverkamp T. Markussen S.. (2011). High coverage sequencing of DNA from microorganisms living in an oil reservoir 2.5 kilometres subsurface. Environ. Microbiol. Rep. 3, 674–681. 10.1111/j.1758-2229.2011.00279.x, PMID: 23761356

[ref31] Lauro F. Bartlett D. (2008). Prokaryotic lifestyles in deep sea habitats. Extremophiles 12, 15–25. 10.1007/s00792-006-0059-5, PMID: 17225926

[ref32] Le Bihan T. Rayner J. Roy M. M. Spagnolo L. (2013). *Photobacterium profundum* under pressure: a MS-based label-free quantitative proteomics study. PLoS One 8:e60897. 10.1371/journal.pone.0060897, PMID: 23741291 PMC3669370

[ref33] Lewin A. Johansen J. Wentzel A. Kotlar H. K. Drabløs F. Valla S. (2014). The microbial communities in two apparently physically separated deep subsurface oil reservoirs show extensive DNA sequence similarities. Environ. Microbiol. 16, 545–558. 10.1111/1462-2920.12181, PMID: 23827055

[ref34] Magot M. (2005). “Indigenous microbial communities in oil fields” in Petroleum microbiology. ed. Magot B. O. A. M. (Washington, DC: ASM Press), 21–33.

[ref35] Magot M. Ollivier B. Patel B. K. (2000). Microbiology of petroleum reservoirs. Antonie Van Leeuwenhoek 77, 103–116. 10.1023/a:1002434330514, PMID: 10768470

[ref36] Martin D. D. Bartlett D. H. Roberts M. F. (2002). Solute accumulation in the deep-sea bacterium *Photobacterium profundum*. Extremophiles 6, 507–514. 10.1007/s00792-002-0288-1, PMID: 12486460

[ref37] Martini S. Al Ali B. Garel M. Nerini D. Grossi V. Pacton M.. (2013). Effects of hydrostatic pressure on growth and luminescence of a moderately-piezophilic luminous bacteria *Photobacterium phosphoreum* ANT-2200. PLoS One 8:e66580. 10.1371/journal.pone.0066580, PMID: 23818946 PMC3688590

[ref38] Ollivier B. Borgomano J. Oger P. (2014). “Petroleum: from formation to microbiology” in Microbial life in the deep biosphere. ed. Wagner J. K. A. D. (GmbH, Berlin/Boston: De Gruyter), 161–185.

[ref39] Ollivier B. Cayol J. -L. (2005). “Fermentative, iron-reducing, and nitrate-reducing microorganisms” in Petroleum microbiology. ed. Magot B. O. A. M. (Washington, DC: ASM Press), 71–88.

[ref40] Picard A. Daniel I. (2013). Pressure as an environmental parameter for microbial life—a review. Biophys. Chem. 183, 30–41. 10.1016/j.bpc.2013.06.019 23891571

[ref41] Pradel N. Ji B. Gimenez G. Talla E. Lenoble P. Garel M.. (2013). The first genomic and proteomic characterization of a deep-sea sulfate reducer: insights into the piezophilic lifestyle of *Desulfovibrio piezophilus*. PLoS One 8:e55130. 10.1371/journal.pone.0055130, PMID: 23383081 PMC3559428

[ref42] Ravot G. Magot M. Fardeau M. L. Patel B. K. Prensier G. Egan A. (1995). *Thermotoga elfii* sp. nov., a novel thermophilic bacterium from an African oil-producing well. Int. J. Syst. Bacteriol. 45, 308–314. 10.1099/00207713-45-2-308 7537064

[ref43] Rees G. N. Harfoot C. G. Sheehy A. J. (1998). Amino acid degradation by the mesophilic sulfate-reducing bacterium desulfobacterium vacuolatum. Arch. Microbiol. 169, 76–80. 10.1007/s002030050543, PMID: 9396838

[ref44] Selinummi J. Seppälä J. Yli-Harja O. Puhakka J. A. (2005). Software for quantification of labeled bacteria from digital microscope images by automated image analysis. BioTechniques 39, 859–863. 10.2144/000112018, PMID: 16382904

[ref45] Simonato F. Campanaro S. Lauro F. M. Vezzi A. D’Angelo M. Vitulo N.. (2006). Piezophilic adaptation: a genomic point of view. J. Biotechnol. 126, 11–25. 10.1016/j.jbiotec.2006.03.038, PMID: 16780980

[ref46] Takai K. Horikoshi K. (2000). *Thermosipho japonicus* sp. nov., an extremely thermophilic bacterium isolated from a deep-sea hydrothermal vent in Japan. Extremophiles 4, 9–17. 10.1007/s007920050002, PMID: 10741832

[ref47] Tamburini C. Boutrif M. Garel M. Colwell R. R. Deming J. W. (2013). Prokaryotic responses to hydrostatic pressure in the ocean—a review. Environ. Microbiol. 15, 1262–1274. 10.1111/1462-2920.12084, PMID: 23419081

[ref48] Tamegai H. Ota Y. Haga M. Fujimori H. Kato C. Nogi Y.. (2011). Piezotolerance of the respiratory terminal oxidase activity of the piezophilic *Shewanella violacea* DSS12 as compared with non-piezophilic *Shewanella* species. Biosci. Biotechnol. Biochem. 75, 919–924. 10.1271/bbb.100882, PMID: 21597190

[ref49] Tanet L. Tamburini C. Baumas C. Garel M. Simon G. Casalot L. (2019). Bacterial bioluminescence: light emission in *Photobacterium phosphoreum* is not under quorum-sensing control. Front. Microbiol. 10:365. 10.3389/fmicb.2019.00365, PMID: 30886606 PMC6409340

[ref50] Van Wambeke F. (1988). Enumeration and size of planktonic bacteria determined by image analysis coupled with epifluorescence. Ann. Inst. Pasteur Microbiol. 139, 261–272. 10.1016/0769-2609(88)90011-7, PMID: 3408594

[ref51] Vigneron A. Alsop E. B. Lomans B. P. Kyrpides N. C. Head I. M. Tsesmetzis N. (2017). Succession in the petroleum reservoir microbiome through an oil field production lifecycle. ISME J. 11, 2141–2154. 10.1038/ismej.2017.78, PMID: 28524866 PMC5563965

[ref52] Yang D. C. Blair K. M. Salama N. R. (2016). Staying in shape: the impact of cell shape on bacterial survival in diverse environments. Microbiol. Mol. Biol. Rev. 80, 187–203. 10.1128/MMBR.00031-15, PMID: 26864431 PMC4771367

[ref53] Yano Y. Nakayama A. Ishihara K. Saito H. (1998). Adaptive changes in membrane lipids of barophilic bacteria in response to changes in growth pressure. Appl. Environ. Microbiol. 64, 479–485. 10.1128/AEM.64.2.479-485.1998, PMID: 16349499 PMC106069

[ref54] Yayanos A. A. (1995). Microbiology to 10,500 meters in the deep sea. Annu. Rev. Microbiol. 49, 777–805. 10.1146/annurev.mi.49.100195.004021, PMID: 8561479

[ref55] Yayanos A. Delong E. (1987). “Deep-sea bacterial fitness to environmental temperatures and pressure” in Current perspectives in high pressure biology. eds. Jannasch H. W. Marquis R. E. Zimmerman A. M. (Toronto: Academic), 17–32.

[ref56] Zobell C. E. Johnson F. H. (1949). The influence of hydrostatic pressure on the growth and viability of terrestrial and marine bacteria. J. Bacteriol. 57, 179–189. 10.1128/JB.57.2.179-189.1949, PMID: 16561663 PMC385493

