# Supplementary material for: Responses to the Hydrostatic Pressure of Surface and Subsurface Strains of Pseudothermotoga elfii Revealing the Piezophilic Nature of the Strain Originating From an Oil-Producing Well
Source: Front Microbiol. 2020 Dec 4;11:588771. doi: 10.3389/fmicb.2020.588771 (PMC7746679; doi:10.3389/fmicb.2020.588771)
Supplement: Supplementary file 1 [file Data_Sheet_1.pdf]

SUPPLEMENTARY MATERIAL

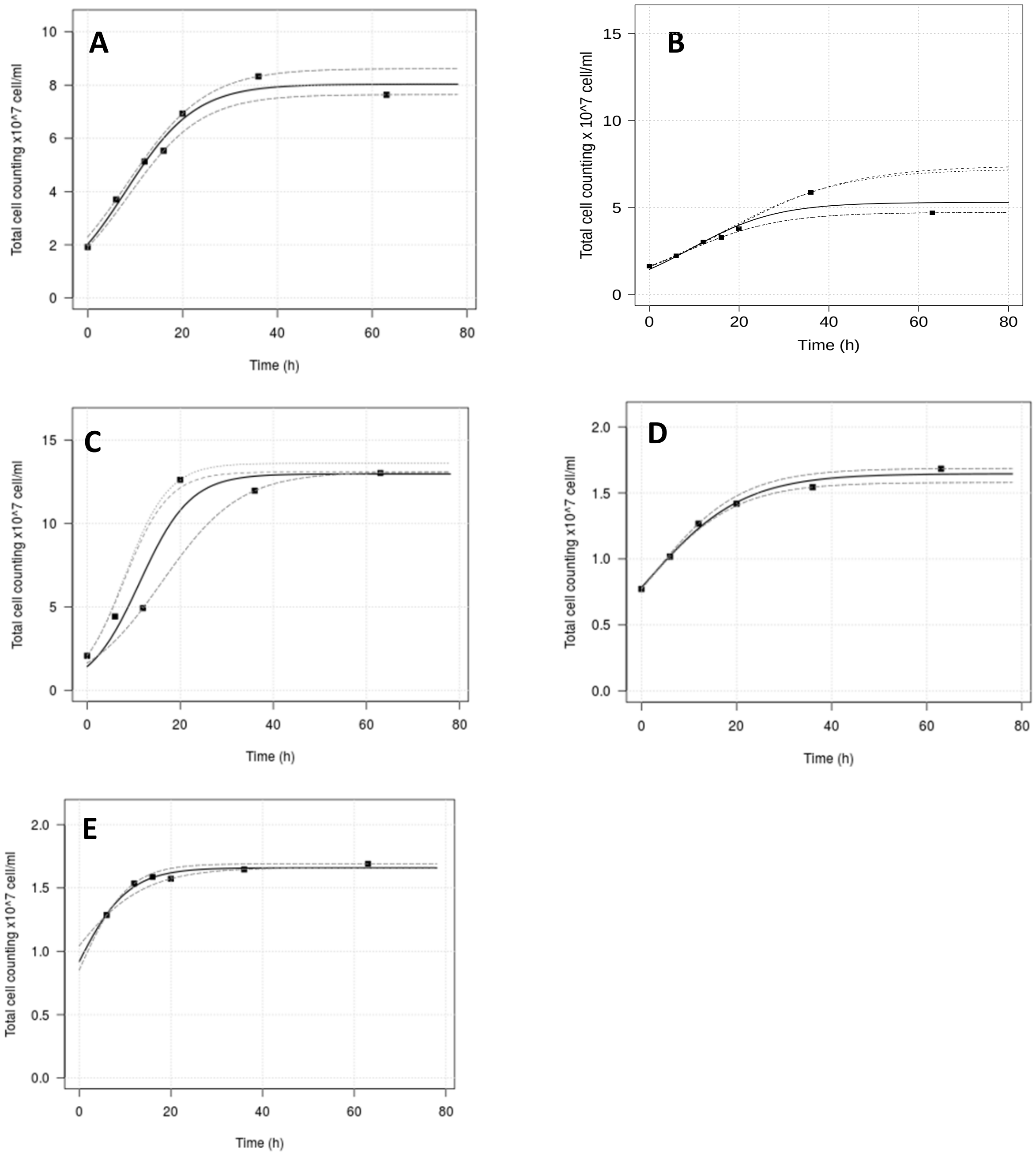

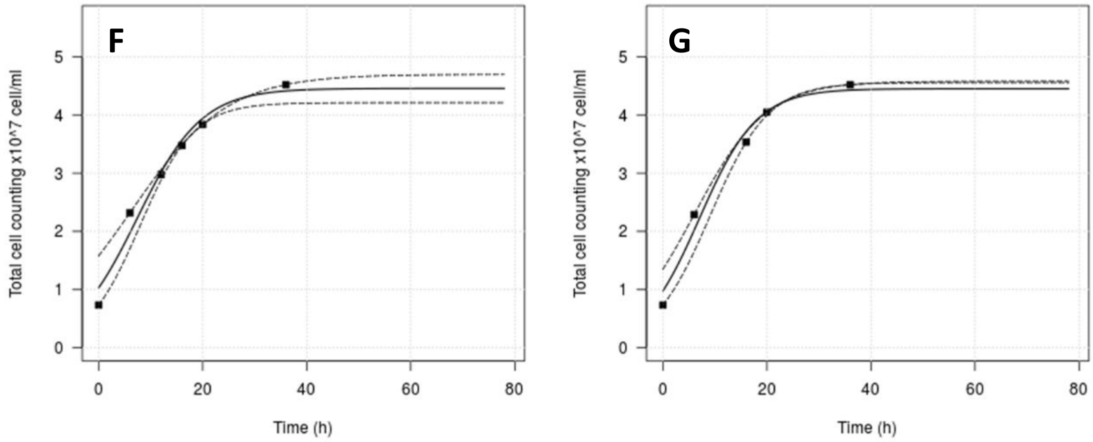

<u>Supplementary figure 1</u>: Growth kinetics of *P. elfii* DSM9442 at 0.1 MPa (A), 10 MPa (B), 20 MPa (C), 30 MPa (D), 40 MPa (E) and of *P. elfii* subsp. *lettingae* at 0.1 MPa (F) and 20 MPa (G). Dot lines are 95% confident interval. Dash lines are 1st and 3rd quartile.

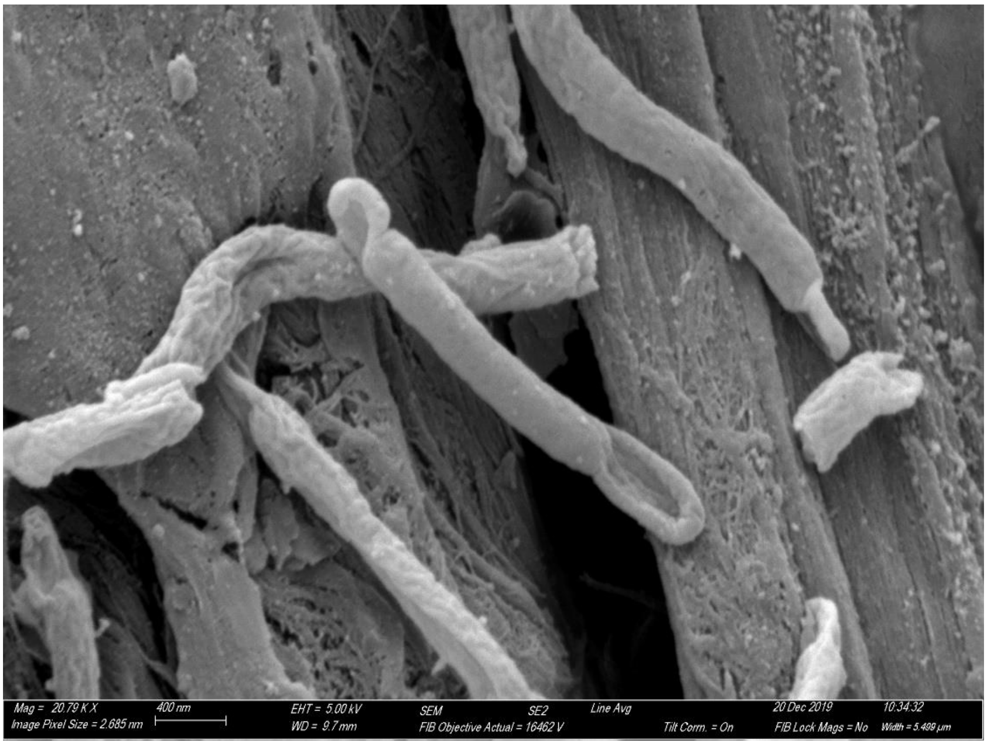

<u>Supplementary figure 2</u>: SEM photography of *P. elfii* DSM9442 grown at 0.1 MPa. Cells were resuspended in a solution composed of 25 mM lysine, 4% paraformaldehyde (PFA), and 2.5% glutaraldehyde (GA) in 0.1 M CA buffer (pH 7.4) and left to soak for 2 hours, undisturbed. Samples were then transferred to a solution of 2.5% GA in 0.1 M CA buffer for 24 hours. Stable, cross-linked samples were rinsed with 0.1 M CA prior to sequential dehydration protocol. Sequential dehydration was conducted using incrementally graded concentrations of acetone in DIH2O, starting with 40%, 50%, 75%, 85%, 95%, and three times at 100%. At each concentration, the sample was left for 20 minutes. Chemical drying was used to complete dehydration by first soaking samples in a 1:1 solution of hexamethyldisilazane:ethanol for 20 minutes. Finally, dehydration was completed by soaking the samples 2 times (20 minutes/time) in 100% hexamethyldisilazane (HMDS). The cell/HMDS mixture was deposited on a gold coated 0.1 µm ceramic filter via vacuum filtration and left to dry for 2 hours prior to sputter coating with gold. Images were acquired with a Zeiss 540 Crossbeam FIB/FESEM.

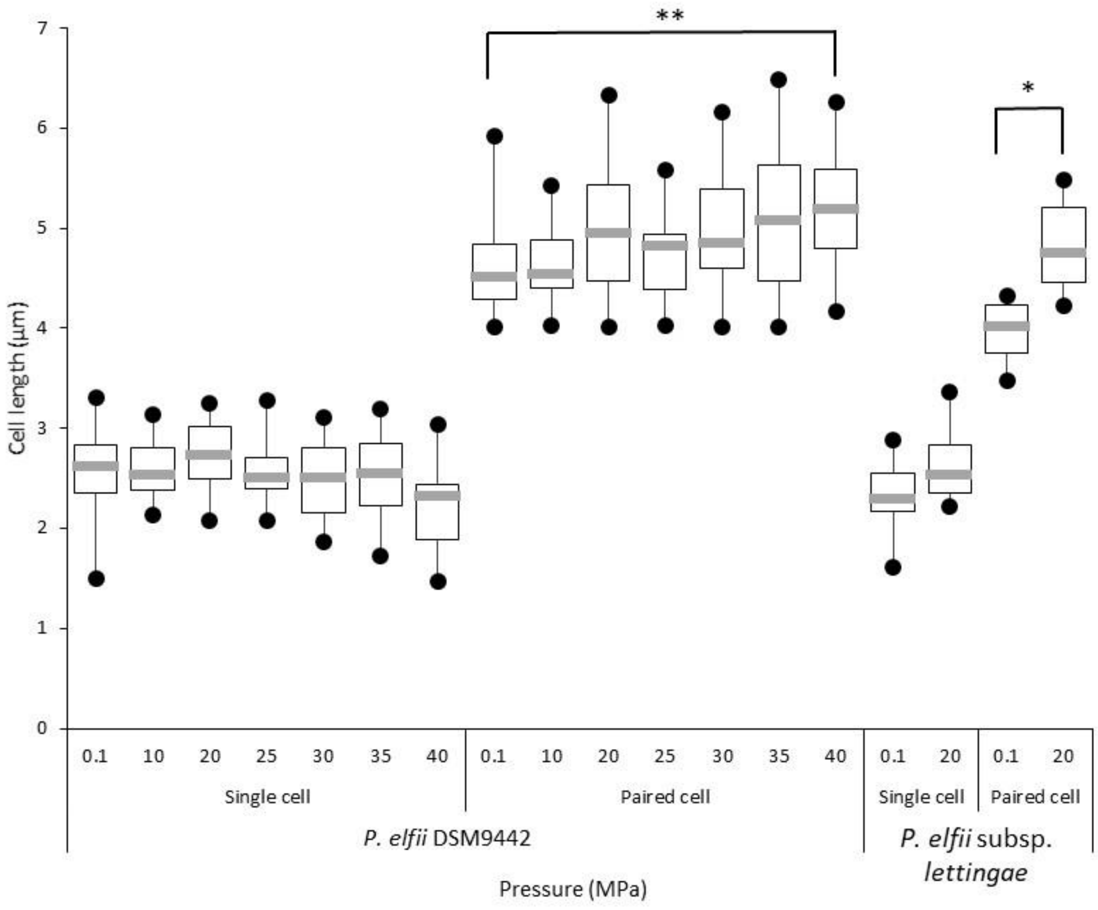

Supplementary figure 3: Size distribution of *P. elfii* DSM9442 and *P. elfii* subsp. *lettingae* cells cultured at various hydrostatic pressures. Box and whiskers plot representing the distribution of the cell length of *P. elfii* DSM9442 and *P. elfii* subsp. *lettingae* cultured at 0.1, 10, 20, 25, 30, 35, 40 MPa and 0.1, 20 MPa, respectively. Both single and paired cells length distribution are presented. Cell length was determined from at least 25 individual items. Significant difference in the cell length are indicated with two asterisk (p<0.0001, α=0.01, Kruskal-Wallis test) and with one asterisk (p<0.0001, α=0.01, Mann-Whitney test). The length of the cells in between the other samples does not significantly differ (p>0.01)

A B

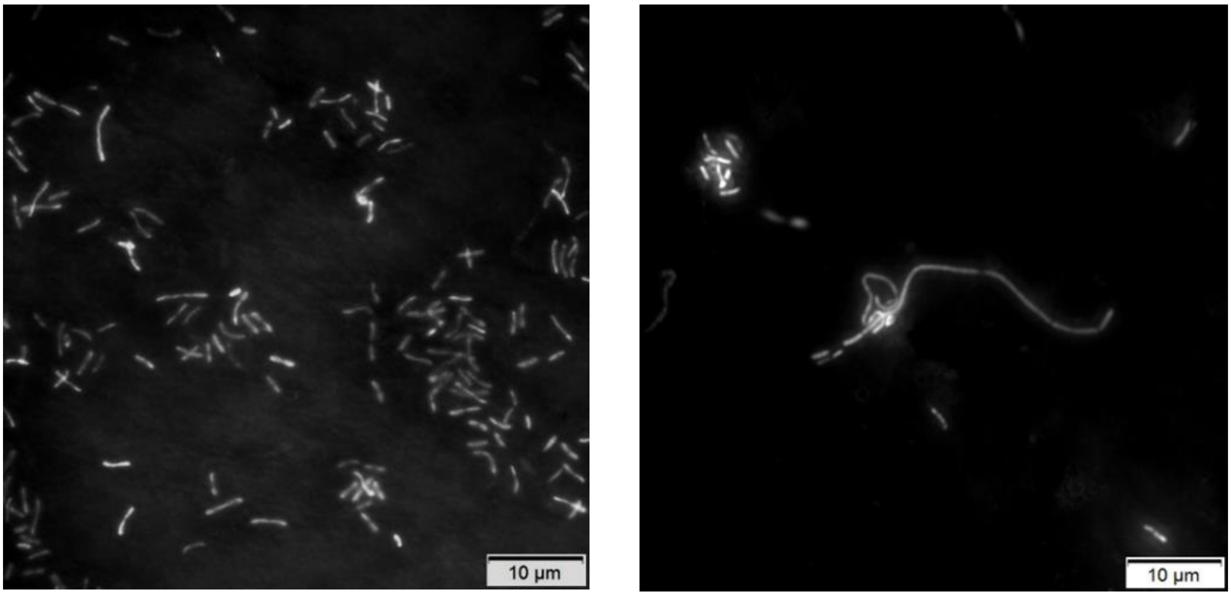

<u>Supplementary figure 4</u>: Phase contrast microscopy of *P. elfii* DSM9442 cultures in the stationary phase after growth at 0.1 MPa (A) and 40 MPa (B).

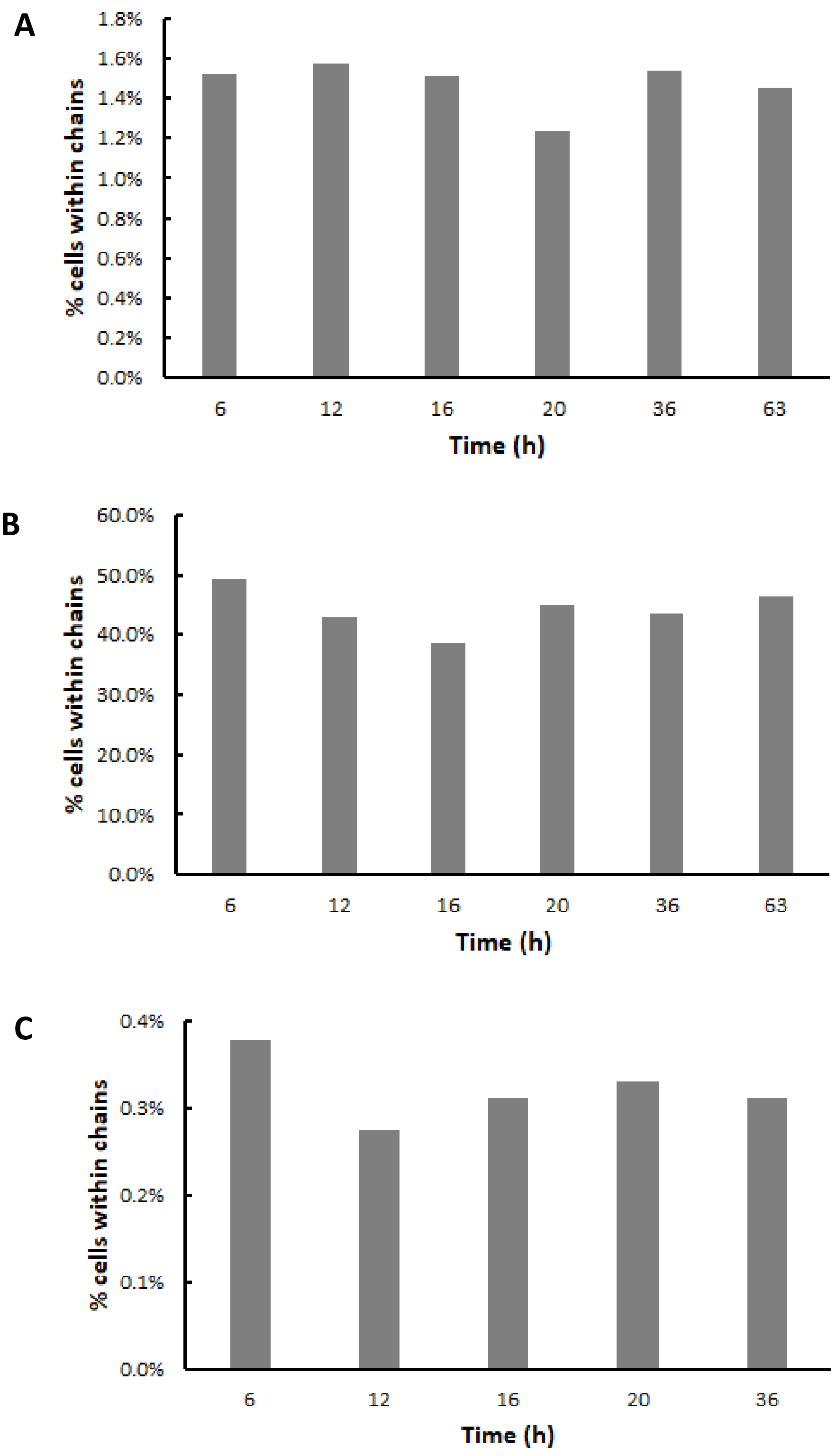

Supplementary figure 5 : Percentage of chained cells in *P. elfii* DSM9442 cultured at 20 MPa (A) and 40 MPa (B), and in *P. elfii* subsp. *lettingae* cultured at 20 MPa (C) at various time of incubation.

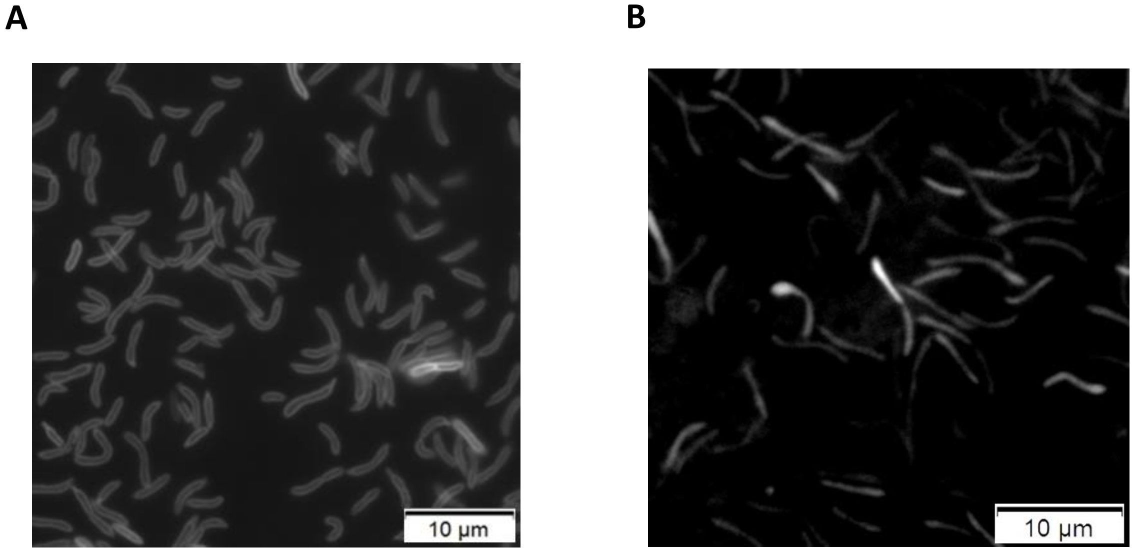

<u>Supplementary figure 6</u>:: Fluorescent microscope images of *P. elfii* DSM9442 cultures after Live/Dead Red staining showing live cells (A) and dead cells (B).
